# Developing a novel paper-based enzymatic biosensor assisted by digital image processing and first-order multivariate calibration for rapid determination of nitrate in food samples

**DOI:** 10.1039/c8ra02792g

**Published:** 2018-06-27

**Authors:** Ali R. Jalalvand, Majid Mahmoudi, Hector C. Goicoechea

**Affiliations:** Research Center of Oils and Fats, Kermanshah University of Medical Sciences Kermanshah Iran ali.jalalvand1984@gmail.com +988334279745 +988334302345; Laboratorio de Desarrollo Analítico y Quimiometría (LADAQ), C_atedra de Química Analítica I, Universidad Nacional del Litoral Ciudad Universitaria, CC 242 (S3000ZAA) Santa Fe Argentina

## Abstract

For the first time, a novel analytical method based on a paper based enzymatic biosensor assisted by digital image processing and first-order multivariate calibration has been reported for rapid determination of nitrate in food samples. The platform of the biosensor includes a piece of Whatman filter paper impregnated with Griess reagent (3-nitroaniline, 1-naphthylamine and hydrochloric acid) and nitrate reductase. After dropping a distinct volume of nitrate solution onto the biosensor surface, nitrate reductase selectively reduces nitrate to nitrite and then the Griess reagent selectively reacts with nitrite to produce a red colored azo dye. Therefore, the color intensity of the produced azo dye is correlated with nitrate concentration. After image capture, the images were processed and digitized in the MATLAB environment by the use of an image processing toolbox and the vectors produced by the digital image processing step were used as inputs of the first-order multivariate calibration algorithms. Several multivariate calibration algorithms and pre-processing techniques have been used to build multivariate calibration models for verifying which technique offers the best predictions towards nitrate concentrations in synthetic samples and the best algorithm has been chosen for nitrate determination in potato, onion, carrot, cabbage and lettuce samples as real cases.

## Abstract 2

For the first time, a novel analytical method based on a paper based enzymatic biosensor assisted by digital image processing and first-order multivariate calibration has been reported for rapid determination of nitrate in food samples.

## Graphical abstract

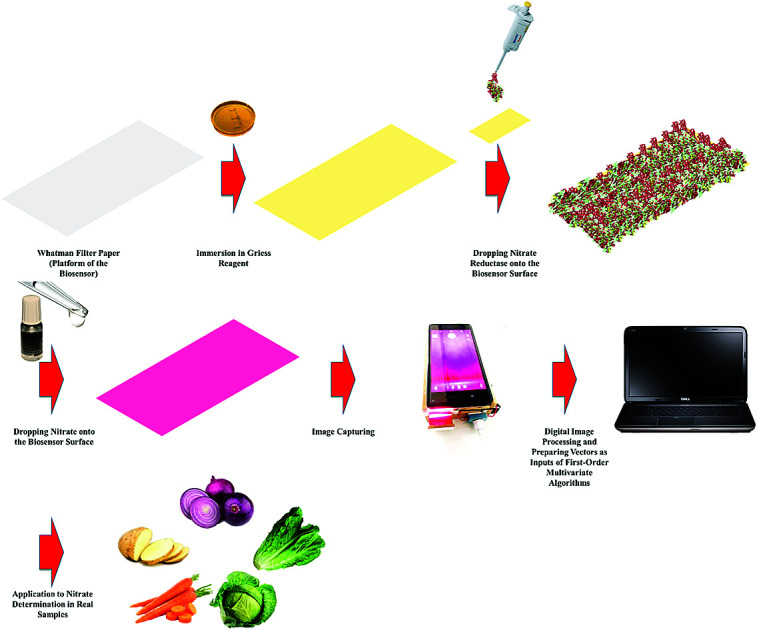

## Introduction

Nitrate is a polyatomic ion which is mainly used as a fertilizer in agriculture.^1^ Nitrate toxicosis occurs by enterohepatic metabolism of nitrate to nitrite and nitrite can oxidize Fe^2+^ to Fe^3+^ in hemoglobin which is then unable to carry oxygen.^2^ This process can cause lack of oxygen in organs and a dangerous condition called methemoglobinemia occurs. In infants, nitrate metabolizing triglycerides are present at higher concentrations relative to other stages of development; therefore, they are especially vulnerable to methemoglobinemia. It should be noted that the acceptable daily intake for nitrate ions has been recommended in the range of 0–3.7 mg (kg body weight)^−1^ day^−1^ by the Food and Drug Administration/World Health Organization (FDA/WHO).^3^ Therefore, development of sensitive analytical methods for nitrate determination is important for controlling the quality of foods.

Some analytical methods have been developed for determination of nitrate such as high-performance liquid chromatography (HPLC/UV),^4^ spectrophotometry,^5^ electrophoresis,^6^ gas chromatography-mass spectroscopy (GC-MS), liquid chromatography (LC),^7^ photometrical method,^8^ potentiometric sensor,^9^ and electrochemical methods.^10–15^ Most of these techniques are expensive and indirect, handling of them needs highly trained technicians and sophisticated instruments. Therefore, developing novel and simple analytical methods which are sensitive, selective, and low cost for nitrate determination is needed.

Chemometrics has obtained widespread applications over the recent decades because of the need to studying complex samples by improving the existing analytical methods. According to the IUPAC definition, calibration is an operation by which an output quantity is related to an input quantity and also called univariate calibration. The input quantities are the concentrations of the analyte of interest and the output quantities are the responses of an analytical instrument. The multivariate calibration methods are widely used to extract information from different types of analytical data to predict the concentrations of the analyte of interest.

Recently, paper based biosensors (PBBs) have received a lot of attention because they are simple, low cost, sensitive, selective and need minimal use of supporting equipment, small volumes of sample, and no external power source since fluidic movement is controlled by capillarity. Therefore, these advantages motivated us to develop a novel enzymatic PBB for determination of nitrate in food samples. Nitrate reductase is a molybdoenzyme which can selectively reduce nitrate to nitrite.^16^ Griess reagent which includes 3-nitroaniline, 1-naphthylamine and hydrochloric acid can selectively react with nitrite to produce a red colored azo dye.^17^ Therefore, in the presence of nitrate reductase and Griess reagent, nitrate is reduced to nitrite and subsequently nitrite can react with Griess reagent to produce a red colored azo dye and the color intensity is proportional to nitrate concentration. In other words, the color change produced at the biosensor surface due to contact with nitrate can be detected and calibrated with nitrate concentration to build a calibration curve which can be used to predict nitrate concentration in unknown samples. Since detecting the color change with the naked eye is difficult, simple systems like digital cameras can be used. In PBB, concentration and some factors such as pH and light can influence the color intensity therefore, multivariate calibration can be a good choice to overcome the mentioned multivariate problem. In the recent years, multivariate calibration methods applied to different types of data are gaining widespread attention for the analysis of complex mixtures.^18–24^ Multivariate calibration techniques which make a relationship between the concentrations of various analytes and multiple measured responses have the advantage of using the full information and not only a characteristic value. Moreover, they allow us a fast determination of components with no prior separation.

**Scheme 1:**
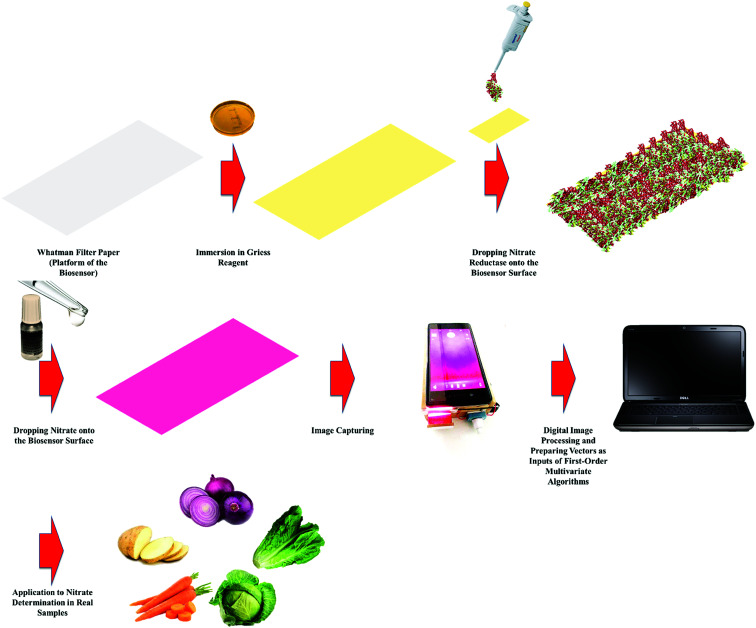
Schematic representation of the proposed methodology employed to determination of nitrate in food samples.

In this work, we developed a novel analytical methodology for nitrate determination based on an enzymatic PBB assisted by digital image processing and first-order multivariate calibration. After capturing the images, they will be processed in MATLAB environment to prepare the inputs of multivariate algorithms and then several multivariate calibration models will be constructed with the help of several algorithms to identify which one offers the best predictions. Finally, the best multivariate calibration model will be applied to determination of nitrate in food samples. Schematic representation of the proposed methodology employed to determination of nitrate in food samples is shown in Scheme 1.

## Experimental

### Chemicals and solutions

All the chemicals used in this study were of analytical reagent grade from regular sources and doubly distilled water (DDW) was used to prepare all the solutions. Sodium nitrate, sodium hydroxide, hydrochloric acid and ethanol were supplied by Merck. Nitrate reductase, 3-nitroaniline and 1-naphthylamine were purchased from Sigma. A stock 0.1 M solution of sodium nitrate was prepared by exact weighing and dissolution of its solid powder in DDW and nitrate working solutions were prepared by diluting the stock solution to appropriate volumes. The Griess reagent including 3-nitroaniline (2.5 × 10^−3^ M), 1-naphthylamine (2.5 × 10^−2^ M) and hydrochloric acid (1.5 × 10^−4^ M) was prepared in DDW/ethanol mixture (50 : 50, v/v%).

### Image capturing system

The image capturing system consists of a smartphone (SONY XPERIA Z5 equipped with a camera (23 MP)), a sample holder to embed the biosensor just under the camera of the smartphone and three LED lamps which provide enough light for sample holder medium.

### Fabrication of the enzymatic PBB

Rectangular pieces of the Whatman filter paper were cut to prepare the platform of the biosensor. Then, the papers were immersed into the Griess reagent including 3-nitroaniline (2.5 × 10^−3^ M), 1-naphthylamine (2.5 × 10^−2^ M) and hydrochloric acid (1.5 × 10^−4^ M) prepared in DDW/ethanol mixture (50 : 50, v/v%) and allowed to be dried at room temperature. Finally, nitrate reductase with a concentration of 10 U mL^−1^ was micropipetted onto the sensor surface.

### Preparation of real samples

To extract nitrate from potato, onion, carrot, cabbage and lettuce samples, certain amounts of crushed samples were left in 150 mL deionized water at 70 °C under stirring for 10 min and then, the remained liquids were filtered.^25^ For nitrate determination in real samples by the proposed methodology in this work, distinct volumes (100 μL) of the solution prepared from real samples were dropped on the PBBs and then their images were captured. To verify the performance of the developed methodology towards nitrate determination in real samples, the real samples were also analyzed by HPLC (KNAUER) with an ODS column (length 250 mm, internal diameter 4.6 mm and particle diameter 5 μm) and a UV detector.

### Mathematical analysis of data

Since several variables such as heterogeneity in the Griess reagent or nitrate reductase deposition on the biosensor surface, and variation in light sources other than nitrate concentration can affect the color intensity and may vary image to image therefore, multivariate calibration is necessary to model their contributions in signal acquisition. Before dropping nitrate solution onto the biosensor surface, we captured an image from its surface to prepare a blank which could be used to correct the color intensity of the images of the samples by subtracting its matrix from the matrices of them. After capturing the images, they were transferred into MATLAB environment to obtain their arrays which could be further processed by the existing commands. Each image in command window is a 2160 × 3840 × 3 array which contains three matrices with size 2160 × 3840 related to red (R), green (G) and blue (B) color intensities, respectively. The array of blank was subtracted from the array of each sample for background elimination by which corrected arrays will be obtained. Then, a matrix with size 2160 × 3840 related to the intensity of red color of each corrected array was extracted and converted to a vector (size 1 × 3840) and subsequently normalized by the use of existing commands. Finally, the normalized vectors obtained from the images were used as inputs of the first-order multivariate algorithms. Partial least squares-1 (PLS-1) and its preprocessing methods such as auto-scaling (ASC) and mean centering (MC) were performed using PLS-Toolbox (Version 3.5, Eigenvector Research Inc., USA,^26^). Computations based on continuum power regression (CPR), robust median centering (RMC), robust L1-median centering (RLMC), partial robust M-regression (PRM), and robust continuum regression (RCR) were performed in MATLAB environment.^27,28^ Computations based on successive projection algorithm (SPA) and multiple linear regression (MLR) were performed in MATLAB environment.^29^ All the computations related to digital image processing and first-order multivariate calibration have been performed in MATLAB environment, Version 7.5.

### Model optimization

To compare multivariate calibration models, the efficiency of the best possible model must be found. Because of a highly correlation between the calibration model efficiency and its parameters, the following parameters must be optimized:

PLS-1: number of latent variables (LVs).

CPR: number of LVs, and power parameter (PP).

PRM: number of LVs, and percentage of data contamination (PDC).

RCR: number of LVs, PDC, PDC and delta parameter (*δ*).

MLR: number of LVs.

### Model efficiency estimation

To verify a model is able to the analysis of real samples or not, validation of the model is an important step in multivariate calibration model building. To achieve this goal, each model was validated for prediction of the concentrations of the validation set by evaluating root mean squared errors of cross-validation (RMSECV), relative error of prediction (REP), root mean square errors of prediction (RMSEP), and cross-validated correlation coefficient (*Q*^2^).1
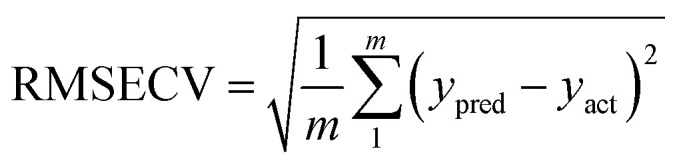
2
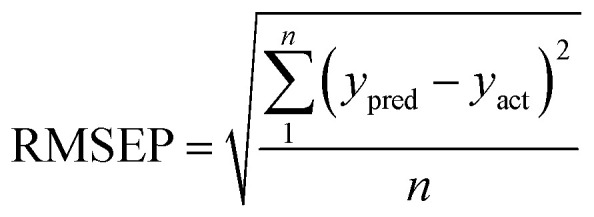
3
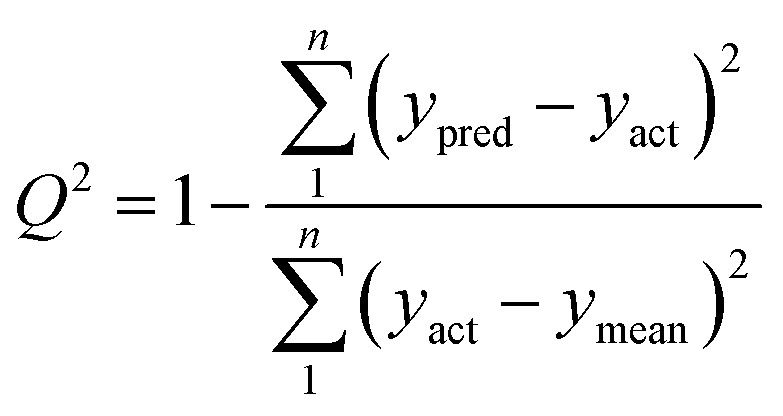
4
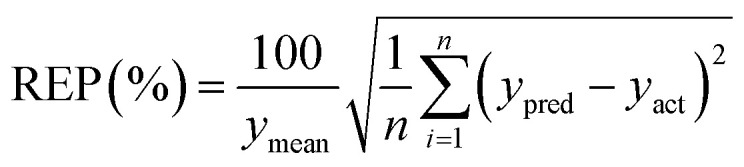
where *y*_act_ and *y*_pred_ are actual and predicted concentrations, respectively, and *y*_mean_ is the mean of the actual concentrations. *m* and *n* are the number of samples in calibration and validation sets, respectively. *R*^2^ is another criterion which could be defined as:5
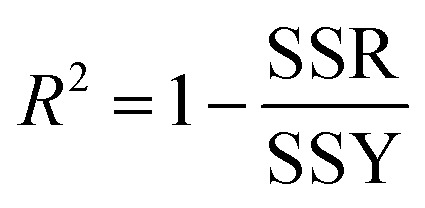
where SSR and SSY are the sum of squares of the residual and the sum of squares, respectively.

## Results and discussion

### Principles of nitrate biosensing

**Scheme 2:**
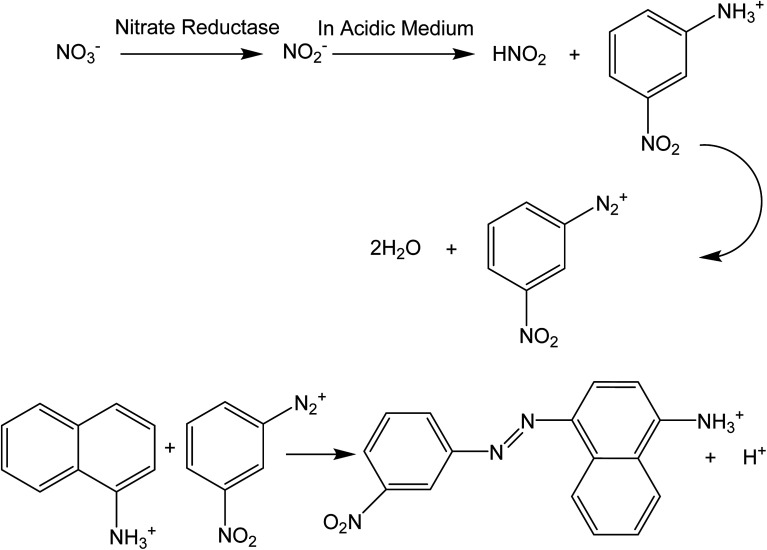
The proposed mechanism for biosensing of nitrate by the developed biosensor in this study.

The platform of the biosensor includes a piece of Whatman filter paper impregnated with Griess reagent (3-nitroaniline, 1-naphthylamine and hydrochloric acid) and nitrate reductase. After dropping a distinct volume (100 μL) of nitrate solution onto the biosensor surface, nitrate reductase selectively reduces nitrate to nitrite and then the Griess reagent selectively reacts with nitrite to produce a red colored azo dye (see Scheme 2). Therefore, the color intensity of the produced azo dye is correlated with nitrate concentration.

### Calibration set

**Fig. 1:**
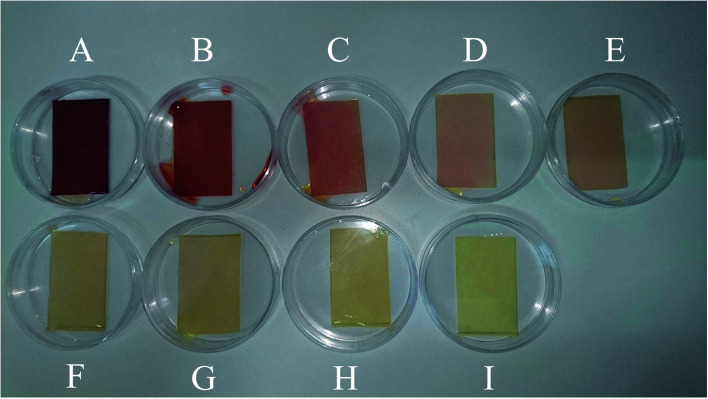
The PBBs contacted with nitrate solutions corresponding to the calibration set: (A) 1.00 × 10^−2^ M, (B) 1.00 × 10^−3^ M, (C) 1.00 × 10^−4^ M, (D) 1.00 × 10^−5^ M, (E) 1.00 × 10^−6^ M, (F) 1.00 × 10^−7^ M, (G) 1.00 × 10^−8^ M, and (H) 1.00 × 10^−9^ M. (I) The PBB without contacting with nitrate solution (blank sample).

**Fig. 2:**
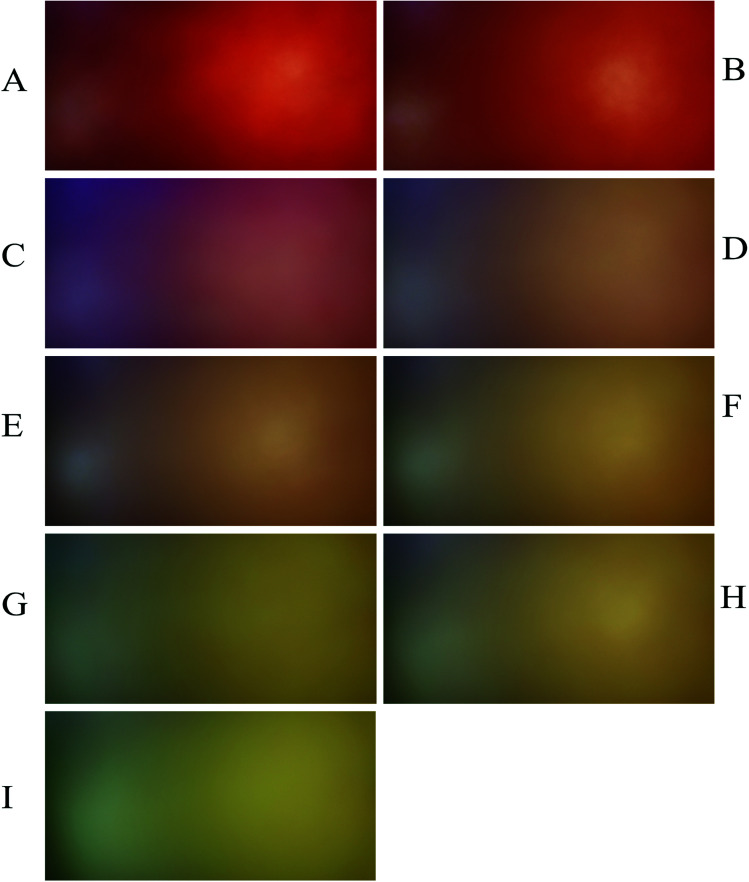
Captured images from the PBBs in the presence of nitrate solutions corresponding to the calibration set: (A) 1.00 × 10^−2^ M, (B) 1.00 × 10^−3^ M, (C) 1.00 × 10^−4^ M, (D) 1.00 × 10^−5^ M, (E) 1.00 × 10^−6^ M, (F) 1.00 × 10^−7^ M, (G) 1.00 × 10^−8^ M, and (H) 1.00 × 10^−9^ M. (I) The captured image from the PBB without contacting with nitrate solution (blank image).

To develop a calibration set, we prepared eight PBBs and after dropping nitrate solutions with concentrations in the range of 1.00 × 10^−9^–1.00 × 10^−2^ M onto them and waiting for 20 minutes, their images were captured. Fig. 1A–H shows the biosensors in contact with nitrate solutions with different concentrations ranging in 1.00 × 10^−9^–1.00 × 10^−2^ M. As can be seen, the color intensity is decreasing with decrease in nitrate concentration. Fig. 2A–H shows the images captured from the surface of the biosensors shown in Fig. 1A–H. In order to correct the color intensities produced by different biosensors, we prepared a PBB without contacting with nitrate solution (Fig. 1I) and then, its image was captured as shown in Fig. 2I. The color intensity of the biosensor shown in Fig. 1I has been subtracted from the color intensity of the all biosensors in contact with nitrate solutions.

### Validation set

**Fig. 3:**
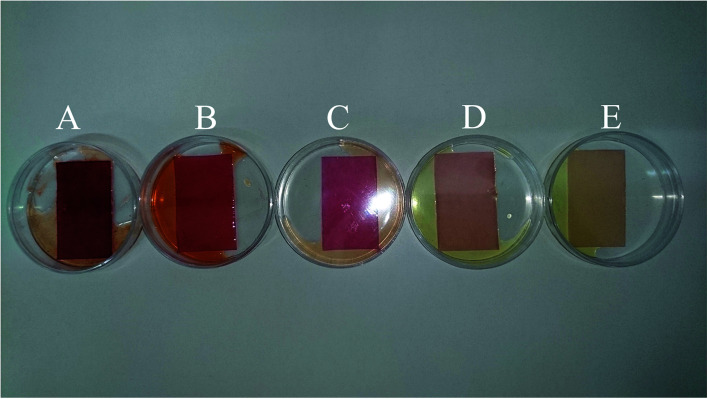
The PBBs in the presence of nitrate solutions corresponding to the validation set: (A) 4.50 × 10^−3^ M, (B) 1.80 × 10^−4^ M, (C) 2.60 × 10^−5^ M, (D) 3.30 × 10^−6^ M, and (E) 1.5 × 10^−6^ M.

**Fig. 4:**
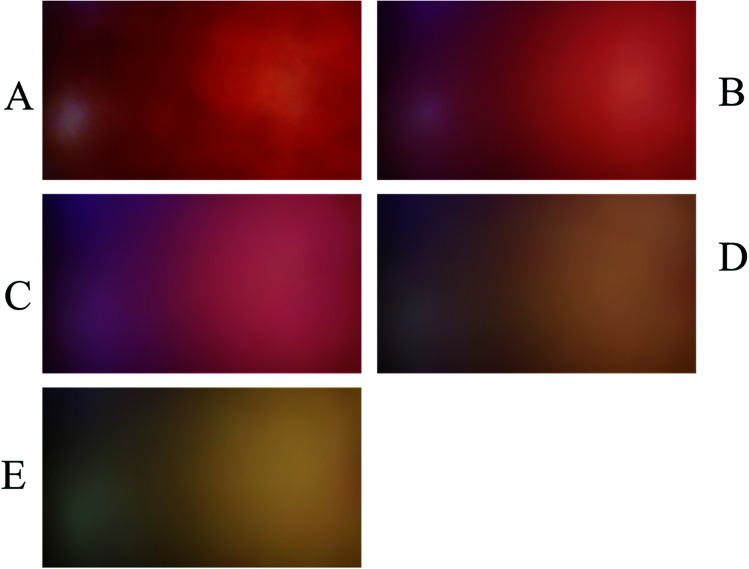
Captured images from the PBBs in the presence of nitrate solutions corresponding to the validation set: (A) 4.50 × 10^−3^ M, (B) 1.80 × 10^−4^ M, (C) 2.60 × 10^−5^ M, (D) 3.30 × 10^−6^ M, and (E) 1.5 × 10^−6^ M.

To evaluate the performance of the developed multivariate calibration models it is necessary to prepare a validation set containing nitrate in the same concentration range used for calibration. Fig. 3A–E shows the PBBs after dropping solutions containing 4.50 × 10^−3^ M, 1.80 × 10^−4^ M, 2.60 × 10^−5^ M, 3.30 × 10^−6^ M and 1.5 × 10^−6^ M nitrate, respectively, onto them. As can be seen, a clear correlation can be observed between the color of the PBBs and nitrate concentration. The images captured from the PBBs mentioned in Fig. 3A–E are shown in Fig. 4A–E. Fig. 4A–E enables us to see the color distribution at the PBBs surface after contacting with nitrate solution.

### Applications of the first-order multivariate calibration algorithms to develop multivariate calibration models

#### PLS-1

The PLS is a famous first-order multivariate calibration methodology which has been frequently applied to different kind of instrumental data with acceptable results.^30,31^ In PLS-1 version, all model parameters will be optimized for quantification of one constituent at a time. In model-training step, an iterative algorithm decomposes the calibration data to correlate the data with concentrations using a so-called inverse model.^32^ This step provides a series of loadings (*p*, size *J* × *A*, *J* is the number of sensors and *A* is the number of LVs), weight-loadings (*W*, size *J* × *A*) and regression coefficients which could be applied to a new sample (*v*, size *A* × 1). Given the profile of an unknown sample *x*_u_ (size *J* × 1), the test sample scores (*t*_u_, for more than one LV, *t*_u_ is a vector) will be obtained by projection of *x*_u_ onto the space of the loadings and weight-loadings:

6 *t*_u_ = (*p*^T^*W*)^−1^*p*^T^*x*_u_

Then, the concentration (*y*_u_) of the analyte in the unknown sample will be estimated by multiplication of *t*_u_ and the regression coefficients:

7 *y*_u_ = *v*^T^*t*_u_

The inputs of PLS-1 were prepared according to the procedure described in Section 2.5. Before calibration, in order to avoid overfitting, the optimum number of LVs were assessed by applying leave one out cross-validation (LOO-CV) method.^31^ Preprocessing methods such as ASC and MC were applied to preprocess the data. The figures of merit obtained for PLS-1, ASC-PLS-1 and MC-PLS-1 are presented in Table 1. As can be seen, by applying preprocessing methods some superiorities can be observed but all the three models didn't show acceptable predictive ability and cannot be recommended for the analysis of real samples.

**Table 1:** Results of applying first-order algorithms to validation samples

Model	LVs^a^	PP^b^	*δ*	PDC^c^	RMSECV	RMSEP	REP (%)	*R* ^2^	*Q* ^2^
PLS-1	3	—	—	—	0.0121	0.0144	15.3069	0.6921	0.7001
MC-PLS-1	2	—	—	—	0.0044	0.0067	7.1529	0.8120	0.8097
ASC-PLS-1	2	—	—	—	0.0033	0.0064	6.8227	0.8199	0.8123
CPR	2	1	—	—	0.0011	0.0062	6.5313	0.8341	0.8411
MC-CPR	1	1	—	—	0.0021	0.0034	3.6328	0.8540	0.8548
ASC-CPR	2	0.5	—	—	0.0088	0.0064	6.8227	0.8500	0.8533
RMC-PRM	1	—	—	10	0.0010	0.0002	0.6004	0.9121	0.9230
RLMC-PRM	1	—	—	10	0.000021	0.00001	0.0102	0.9891	0.9901
RMC-RCR	1	—	0.5	10	0.0038	0.002	2.1321	0.9231	0.9199
RLMC-RCR	1	—	0.5	10	0.0042	0.002	2.1317	0.9188	0.9321
MLR-SPA	1	—	—	—	0.2109	0.1931	204.97	0.5411	0.4830

a Latent variables.

b Power parameter.

c Percentage of data contamination.

#### CPR

No mathematical details of CPR will be described here, for a detailed description of theoretical aspects of CPR, see ref. 33 The MC and ASC were used as preprocessing method, LOO-CV was used for determination of the number of LVs and power parameter was also optimized. The results of application of CPR to validation set are presented in Table 1. According to the values of RMSECV, REP, *R*^2^, RMSEP and *Q*^2^ it can be concluded that CPR is pretty better than PLS-1 but could not be recommended for the analysis of real samples.

#### PRM and RCR

Classical multivariate algorithms are extremely sensitive to the existence of outliers and their performance can decrease in the presence of outliers therefore, the presence of outliers in data used for constructing multivariate calibration models was checked by robust principal component analysis (rPCA).^34^ Here, using robust and robust orthogonal distances a distance–distance plot (not shown) was made which could be used to identify the outliers. For the *i*th sample, the robust distance, RD_*i*_, is defined according to the following equation:8
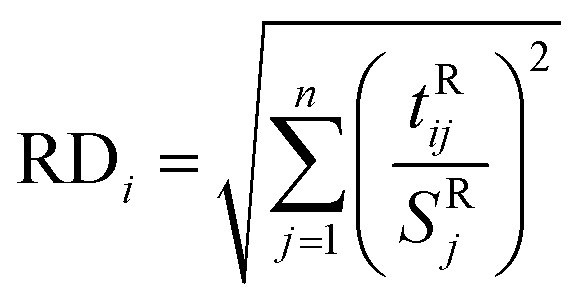
where the elements of the robust score matrix are collected in *t*^R^_*ij*_ and *S*^R^_*j*_ refers to squared root of the *j*th robust eigenvalue. The orthogonal distances could be obtained by the following equation:

9 OD_*i*_ = ‖*x*_*i*_ − *P*_*f*_*t*^T^_*i*,*f*_‖

where *t*_*i*,*f*_ is the *i*th score vector containing *f* elements and *P*_*f*_ is a matrix (*n*,*f*) including *f* robust loadings. For detecting outlying observation z-transformed distances can be used, *i.e.*, to center all the vectors with distances around the median and to divide all elements by corresponding *Q*_*n*_-scale of the distances.^35^ A general cutoff value of 3 was used for z-transformed distances which considers all the objects with z-transformed distances higher than 3 as outliers (here, one outlier was detected).

The presence of the outliers in data used for model building may disrupted the performance of PLS-1 and CPR. Therefore, RCR and PRM as robust algorithms which within an outlier-free subset of the data,^36,37^ were used for predicting concentrations of nitrate in the validation set. RMC and RLMC were used to pre-process the data and determination of the number of LVs was performed by LOO-CV. Maximum percentage of data contamination was fixed at 10. Results of application of RCR and PRM to validation set are presented in Table 1. As can be seen, obvious improvement is notable in the results especially for PRM with RLMC as preprocessing technique. Using a subset of data free from the outlier is a great help for PRM to predict nitrate concentrations in good accordance with nominal ones. But, to make a final decision about choosing the best algorithm for the analysis of real samples, the results of MLR-SPA must also be checked.

#### MLR-SPA

The MLR is the simplest technique for building a calibration model.^38,39^ The collinearity problems could be tackled by SPA as a variable selection technique.^40^ The SPA can also provide simpler models and better predictions. For this purpose, variable selection was performed by SPA for obtain a simpler MLR model. The results of application of MLR-SPA to validation samples are presented in Table 1. According to the results presented in Table 1, MLR-SPA showed a very weak performance to predict nitrate concentrations in validation samples and definitely cannot be recommended for the analysis of real samples.

### Comparison of first-order algorithms

**Fig. 5:**
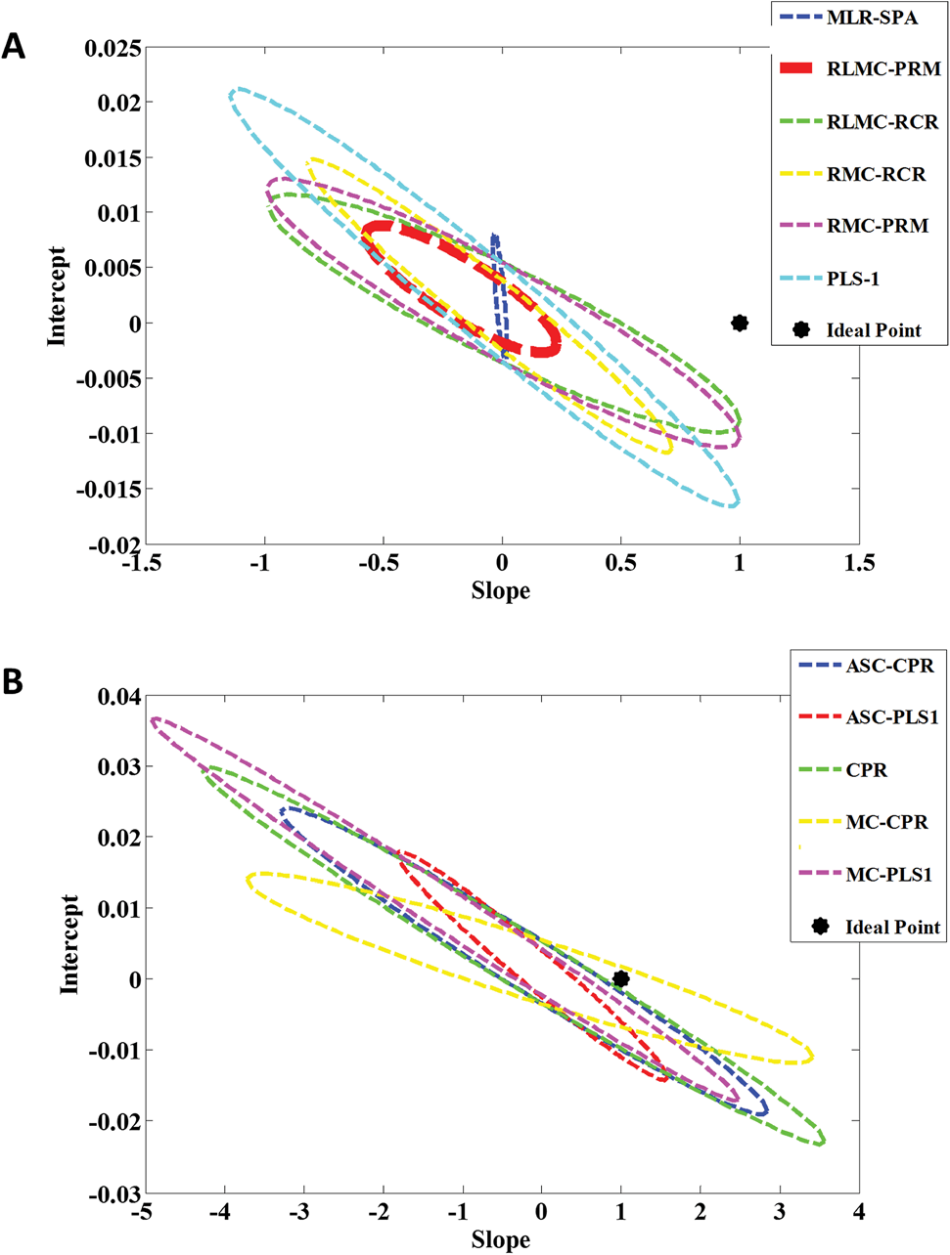
Elliptical joint confidence regions (at 95% confidence level) for the slopes and intercepts of the regressions for predicted nitrate concentrations by (A) MLR-SPA, RLMC-PRM, RLMC-RCR, RMC-RCR, RMC-PRM and PLS-1 and (B) ASC-PLS-1, CPR, MC-CPR and MC-PLS-1 in validation set on their nominal concentrations.

In order to compare accuracy and precision of the first-order multivariate algorithms used in this study, the predicted concentrations related to the validation set were regressed on the actual (nominal) ones by the analysis of predicted concentrations *versus* actual concentrations by ordinary least squares (OLS).^41^ Elliptical joint confidence region (EJCR) test was used to compare the calculated intercept and slope with their expected values (intercept = 0, slope = 1) which is also called the ideal point. Therefore, if the ellipses obtained by EJCR include the ideal point the predicted and nominal values are not significantly different. The size of ellipses refers to the precision of the method, smaller sizes confirm higher precisions.^42^Fig. 5A and B show the EJCRs (at 95% confidence level) for the slopes and intercepts of the regressions for nitrate concentrations in validation set predicted by different first-order multivariate calibration models. As can be seen, the best performance (smaller ellipse which contains the ideal point) is observed for application of RLMC-PRM to predict nitrate concentrations in validation relative to the other algorithms. The statistical results presented in Table 1 also support the results of this section.

### Analysis of real samples for nitrate determination

**Fig. 6:**
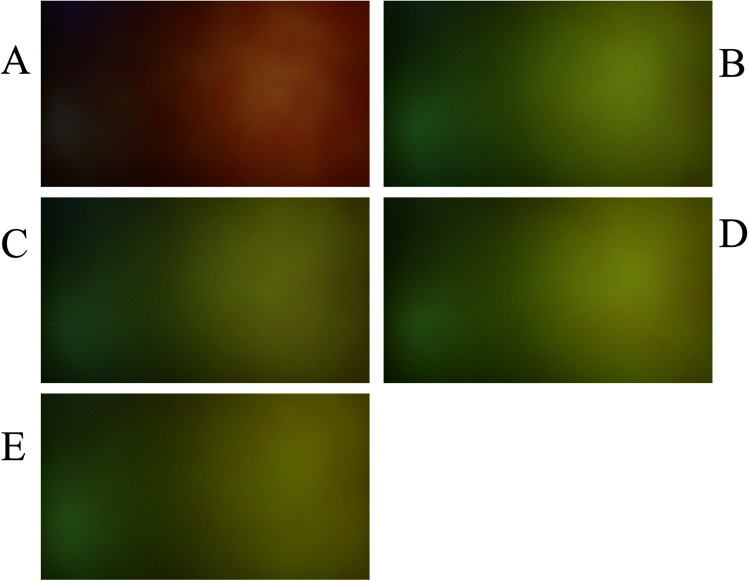
Showing the corrected images of real samples by the imshow command: (A) potato, (B) onion, (C) carrot, (D) cabbage, and (E) lettuce.

After preparing the real samples according to the procedure described in section preparation of real samples, the RLMC-PRM model with optimized parameters was used to estimate nitrate concentration in potato, onion, carrot, cabbage and lettuce as real samples. Because of the probable existence of nitrite in real samples and in order to obtain reliable results for nitrate concentration, each sample is analyzed by two PBBs. At first, a PBB without nitrate reductase (PBB*) is applied and then, an enzymatic PBB which contains nitrate reductase is applied. After capturing the images of the mentioned PBBs, the matrix of the PBB* is subtracted from PBB matrix (corrected matrix of real sample = matrix PBB − matrix PBB*). Finally, the corrected matrices of real samples were converted to vectors and were used as inputs of RLMC-PRM for predicting nitrate concentrations. The corrected matrices of real samples are shown by the imshow command in Fig. 6. The results of the analysis of real samples are presented in Table 2. The *t*-test was used to compare the results of the developed method in this study with those obtained by the reference method (HPLC/UV) and the results are presented in Table 2. Since the experimental values of *t* are less than their critical value (*t* = 3.18, *p* < 0.05) therefore, the null hypothesis is not rejected which confirms that the methods do not give significantly different results for the nitrate concentration.

**Table 2:** Determination of nitrate in real samples by RLMC-PRM and HPLC/UV

Sample	Not spiked samples			Spiked samples				RLMC-PRM (recovery (%))	HPLC/UV (recovery (%))
	RLMC-PRM (found, *n* = 4)	HPLC/UV (found, *n* = 4)	*t* (*p* < 0.05)	Added	RLMC-PRM (found, *n* = 4)	HPLC/UV (found, *n* = 4)	*t* (*p* < 0.05)		
Potato	5.33(±0.08) × 10^−4^ M	5.12(±0.12) × 10^−4^ M	3.05	8.50 × 10^−4^ M	14.01(±0.04) × 10^−4^ M	14.03(±0.02) × 10^−4^ M	3.11	102.07	104.60
Onion	1.12(±0.08) × 10^−8^ M	1.18(±0.04) × 10^−8^ M	2.99	2.30 × 10^−8^ M	3.51(±0.11) × 10^−8^ M	3.31(±0.09) × 10^−8^ M	3.09	103.77	92.60
Carrot	1.19(±0.05) × 10^−8^ M	1.08(±0.10) × 10^−8^ M	3.04	4.70 × 10^−8^ M	5.68(±0.12) × 10^−8^ M	5.83(±0.14) × 10^−8^ M	3.14	95.53	101.05
Cabbage	7.95(±0.07) × 10^−8^ M	7.81(±0.09) × 10^−8^ M	3.01	3.12 × 10^−8^ M	11.11(±0.11) × 10^−7^ M	10.98(±0.04) × 10^−7^ M	3.12	101.26	101.58
Lettuce	1.26(±0.12) × 10^−7^ M	1.41(±0.05) × 10^−7^ M	3.03	5.50 × 10^−7^ M	6.84(±0.06) × 10^−7^ M	6.88(±0.04) × 10^−7^ M	3.10	101.43	99.45

When a new analytical method is developed, it is necessary to verify its performance with that of a reference method. Regarding this important step, we expanded our work for comparing its accuracy towards nitrate determination with HPLC/UV as the reference method. Table 2 shows the results obtained by our method and HPLC/UV. The results of the proposed method are comparable with those of HPLC/UV. Therefore, according to the results obtained in this section, the method described in this study is highly recommended for nitrate determination in food samples.

## Conclusions

In this study, a novel, sensitive, selective and very low-cost analytical method based on an enzymatic biosensor assisted by digital image processing and first-order multivariate calibration was introduced for rapid determination of nitrate in food samples. The proposed biosensor significantly enhances the selectivity towards nitrate ions due to the existence of nitrate reductase and Griess reagent. After reduction of nitrate to nitrite by nitrate reductase, the Griess reagent selectively reacts with nitrite to produce a red colored azo dye and its color intensity is correlated with nitrate concentration. After image capturing, the images were digitized and processed in MATLAB environment and converted to the vectors by the existing commands. The vectors were used to construct multivariate calibration models. After multivariate calibration model development by PLS-1, PRM, CPR, RCR and MLR-SPA their performances towards nitrate determination in synthetic samples were compared and RLMC-PRM was chosen as the best one for the analysis of real samples. Finally, the application of the developed method to assay nitrate concentration in potato, onion, carrot, cabbage and lettuce as real cases allowed us to obtain satisfactory results which were in a good agreement with the HPLC/UV as reference method. This study allowed us to present an analytical method as an efficient, low-cost and accessible alternative for routine determination of nitrate in food samples. Taking into account that applying HPLC/UV to nitrate determination suffers from some problems such as instrumental limitations, cost and time, use of the method described in our study is highly recommended.

## Conflicts of interest

There are no conflicts to declare.

## Supplementary Material

## References

[cit1] Manea F., Remes A., Radovan C., Pode R., Picken S., Schoonman J. (2010). Talanta.

[cit2] https://en.wikipedia.org/wiki/Nitrate

[cit3] Rezaei M., Fani A., Moini A. L., Mirzajani P., Malekirad A. A., Rafiei M. (2014). Int. Math. Res. Notices.

[cit4] Croitoru M. D. (2012). J. Chromatogr. B.

[cit5] Moo Y. C., Matjafri M. Z., Lim H. S., Tan C. H. (2016). Optik.

[cit6] Freitas C. B., Moreira R. C., de Oliveira Tavares M. G., Coltro W. K. T. (2016). Talanta.

[cit7] Akyüz M., Ata S. (2009). Talanta.

[cit8] Tu X., Xiao B., Xiong J., Chen X. (2010). Talanta.

[cit9] Hassan S. S. M., Sayour H. E. M., Al-Mehrezi S. S. (2007). Anal. Chim. Acta.

[cit10] Manea F., Remes A., Radovan C., Pode R., Picken S., Schoonman J. (2010). Talanta.

[cit11] Shiddiky M. J. A., Won M.-S., Shim Y.-B. (2006). Electrophoresis.

[cit12] Afkhami A., Madrakian T., Ghaedi H., Khanmohammadi H. (2012). Electrochim. Acta.

[cit13] Shariar S. M., Hinoue T. (2010). Anal. Sci..

[cit14] Madasamy T., Pandiaraj M., Balamurugan M., Bhargava K., Sethy N. K., Karunakaran C. (2014). Biosens. Bioelectron..

[cit15] Kaminskaya O. V., Zakharova E. A., Slepchenko G. B. (2004). J. Anal. Chem..

[cit16] https://en.wikipedia.org/wiki/Nitrate_reductase

[cit17] Weselsky P., Benedikt R. (1879). Ber. Dtsch. Chem. Ges..

[cit18] Gholivand M. B., Jalalvand A. R., Goicoechea H. C., Skov T. (2014). Talanta.

[cit19] Gholivand M. B., Jalalvand A. R., Goicoechea H. C., Gargallo R., Skov T., Paimard G. (2015). Talanta.

[cit20] Gholivand M. B., Jalalvand A. R., Goicoechea H. C., Skov T. (2015). Talanta.

[cit21] Jalalvand A. R., Gholivand M. B., Goicoechea H. C., Rinnan Å., Skov T. (2015). Chemom. Intell. Lab. Syst..

[cit22] Jalalvand A. R., Gholivand M. B., Goicoechea H. C. (2015). Chemom. Intell. Lab. Syst..

[cit23] Jalalvand A. R., Goicoechea H. C., Rutledge D. N. (2017). TrAC, Trends Anal. Chem..

[cit24] Jalalvand A. R., Goicoechea H. C. (2017). TrAC, Trends Anal. Chem..

[cit25] Bagheri H., Hajian A., Rezaei M., Shirzadmehr A. (2017). J. Hazard. Mater..

[cit26] http://www.eigenvector.com/

[cit27] Walczak B., Massart D. L. (1996). Anal. Chim. Acta.

[cit28] Walczak B., Massart D. L. (1996). Anal. Chim. Acta.

[cit29] Paiva H. M., Carreiro Soares S. F., Harrop Galvão R. K., Ugulino Araújo M. C. (2012). Chemom. Intell. Lab. Syst..

[cit30] Martens H. and Naes T., Multivariate Calibration, Wiley, Chichester, 1989

[cit31] Haaland D. M., Thomas E. V. (1988). Anal. Chem..

[cit32] Haaland D. M., Thomas E. V. (1988). Anal. Chem..

[cit33] de Jong S., Farebrother R. W. (1994). Chemom. Intell. Lab. Syst..

[cit34] Li G., Chen Z. L. (1985). J. Am. Stat. Assoc..

[cit35] Rousseeuw P. J., Croux C. (1993). J. Am. Stat. Assoc..

[cit36] Serneels S., Filzmoser P., Croux C., Van Espen P. J. (2005). Chemom. Intell. Lab. Syst..

[cit37] Serneels S., Croux C., Filzmoser P., Van Espen P. J. (2005). Chemom. Intell. Lab. Syst..

[cit38] Ni Y., Wang L., Kokot S. (2001). Anal. Chim. Acta.

[cit39] Bessant K., Saini S. (2000). J. Electroanal. Chem..

[cit40] Araujo M. C. U., Saldanha T. C. B., Galvão R. K. H., Yoneyama T., Chame H. C., Visani V. (2001). Chemom. Intell. Lab. Syst..

[cit41] Gonzalez A. G., Herrador M. A., Asuero A. G. (1999). Talanta.

[cit42] Arancibia J. A., Escandar G. M. (2003). Talanta.

